# Progress towards the Development of Research Agenda and the Launch of Knowledge Hub: The WHO Thematic Platform for Health Emergency and Disaster Risk Management Research Network (Health EDRM RN)

**DOI:** 10.3390/ijerph18094959

**Published:** 2021-05-07

**Authors:** Ryoma Kayano, Shuhei Nomura, Jonathan Abrahams, Qudsia Huda, Emily Y. Y. Chan, Virginia Murray

**Affiliations:** 1 World Health Organization Centre for Health Development, 1-5-1 Wakinohama-Kaigandori, Chuo-ku, Kobe 651-0073, Japan; 2 Department of Health Policy and Management, School of Medicine, Keio University, 35 Shinanomachi, Shinjuku-ku, Tokyo 160-8582, Japan; s-nomura@keio.jp; 3 Department of Global Health Policy, Graduate School of Medicine, The University of Tokyo, 7-3-1 Hongo, Bunkyo-ku, Tokyo 113-0033, Japan; 4 Disaster Risk Management and Resilience Unit, Health Security Preparedness Department, World Health Organization, CH-1211 Geneva, Switzerland; abrahamsj@who.int (J.A.); hudaq@who.int (Q.H.); 5 Collaborating Centre for Oxford University and CUHK for Disaster and Medical Humanitarian Response (CCOUC), The Chinese University of Hong Kong, Hong Kong, China; emily.chan@cuhk.edu.hk; 6 Public Health England, London SE1 8UG, UK; Virginia.Murray@phe.gov.uk

**Keywords:** health emergency and disaster risk management (health EDRM), health EDRM research network, health EDRM knowledge hub, health EDRM research agenda, WHO thematic platform for health EDRM

## Abstract

In response to the increasing burden of recent health emergencies and disasters, the World Health Organization (WHO) and its partners established the WHO thematic platform for health emergency and disaster risk management research network (health EDRM RN) in 2016, with the purposes of promoting global research collaboration among various stakeholders and enhancing research activities that generate evidence to manage health risks associated with all types of emergencies and disasters. With the strong support and involvement of all WHO regional offices, the health EDRM RN now works with more than 200 global experts and partners to implement its purposes. The 1st and 2nd Core Group Meetings of the health EDRM RN were held on 17–18 October 2019 and 27 November 2020, respectively, to discuss the development of a global research agenda that the health EDRM RN will focus on facilitating, promoting, synthesizing and implementing, taking into account the emergence of the coronavirus disease 2019 (COVID-19) (health EDRM RN research agenda). A focus of the meetings was the establishment of an online platform to share information and knowledge, including the databases that the health EDRM RN accumulates (WHO health EDRM knowledge hub). This paper presents a summary of the discussion results of the meetings.

## 1. Introduction

The number and severity of health emergencies and disasters are increasing as a result of a variety of factors, including unplanned urbanization, climate change and an increasing number of vulnerable elderly people [1,2]. There is an urgent need for evidence-based health emergency and disaster risk management policies and programs through effective prevention, preparedness, response and recovery measures. At the 3rd United Nations World Conference on Disaster Risk Reduction (WCDRR) held in Japan in 2015, the Sendai Framework for Disaster Risk Reduction 2015–2030—including four priority actions and seven targets that cover prevention, preparation, response and recovery for disaster risk management—was formulated and adopted, underpinned by the Sustainable Development Goals (SDGs) [3].

In response, the World Health Organization (WHO) and its partners established the WHO thematic platform for health emergency and disaster risk management research network (health EDRM RN) in 2016 to promote research collaboration worldwide among academia, government officials and other stakeholders, strengthen research activities that generate evidence to manage health risks related to all types of emergencies and disasters, and better inform policy and practice [4,5].

With the strong support and involvement of all WHO regional offices, the health EDRM RN has now promoted global research collaboration and knowledge integration with more than 200 global experts and partner institutions. The current main priorities of the health EDRM RN are (1) setting the research agenda that the health EDRM RN focuses on facilitating, promoting, synthesizing and implementing (i.e., the health EDRM RN research agenda) and (2) establishing an online platform to share information and knowledge, including the databases that the health EDRM RN accumulates (i.e., the WHO health EDRM knowledge hub). Through these actions, the health EDRM RN, guided by the WHO health EDRM framework [6], aims to promote coherence for the implementation of the key outputs of the WHO 13th General Program of Work (GPW 13), the International Health Regulations (IHR), the Sendai Framework, the 2030 Agenda for Sustainable Development, the Paris Agreement on Climate Change, and the health aspects of other relevant global, regional and national frameworks [7]. This paper summarises the discussion results of the 1st and 2nd health EDRM RN core group meetings held on 17–18 October 2019 and 27 November 2020, respectively.

## 2. Materials and Methods

The health EDRM RN core group meeting was organized by the WHO Centre for Health Development (WHO Kobe Centre, Kobe, Japan) for the first time to discuss the key research themes and related research questions of the health EDRM, with 21 experts participating. The second meeting was the update conference, which took place online in the light of the coronavirus disease 2019 (COVID-19) pandemic, involving 33 experts. Participants included stakeholders from the WHO headquarters and all regional offices, as well as external experts. The summaries presented in this paper are the results of the discussions from both meetings. Note that prior to the 1st core group meeting, the WHO health EDRM research agenda was discussed at the expert meeting organized by WHO Kobe Centre on 17 October 2018 (namely, the 2018 Kobe Expert Meeting), with various WHO partners invited including the World Association for Disaster and Emergency Medicine (WADEM) and Japan International Cooperation Agency (JICA), with the summary of this meeting published elsewhere [8].

## 3. Results

### 3.1. WHO Health EDRM Research Agenda

The WHO health EDRM research agenda was primarily the subject of the 1st core group meeting. To address the comprehensive needs of health EDRM research, research priorities were discussed and the need to take an all-hazard approach to natural hazards, disease outbreaks, societal hazards (e.g., conflicts and refugees) and technological hazards in different national contexts was stressed. Participants added three new research themes (translational research, complex health risk conceptualization and a cross-disciplinary research agenda) and associated questions to the five tentative research areas proposed at the 2018 Kobe Expert Meeting [8], focusing on more specific practical and operational needs (Table 1). Note that a part of this table was adapted from a report of the 1st core group meeting issued by the WHO Kobe Centre [9] and Kayano et al. [8]. First, as the interaction between science, policy and practice has been poor traditionally and is in need of improvement, the health ERDM research agenda is expected to work on promoting successful mechanisms to accelerate the translation of research findings into policy and practice. Second, health EDRM research is expected to deal with a wide range of risks and events, including those associated with natural hazards, human-induced hazards and other complex emergencies. The research agenda is expected to show possible directions for conceptualizing the complex health risks associated with different emergencies and disasters. Third, health EDRM operations are expected to be conducted in collaboration with multiple sectors. The research agenda is expected to highlight overlapping research topics with other non-health sectors and possible lessons for effective multi-disciplinary collaboration.

On the other hand, participants agreed that a consensus-building process based on appropriate prioritization criteria for research themes and questions is necessary for the formulation of the WHO health EDRM research agenda, and that strict coordination with stakeholders is required. A mix of several criteria for developing a research agenda, as addressed by the participants, included being (a) fit for purpose, (b) scientifically rigorous, (c) timely, (d) fit for translation for implementation, (e) transparent, (f) ethically sound, (g) useful for multiple sectors, (h) valid for multiple types of risks/hazardous events/disasters, (i) equipped with high political and social impact, and (j) a benefit for countries, as reported by the WHO Kobe Centre [9]. These are preliminary criteria, which will be further reviewed and finalized, accompanied by the rationale for each criterion. Participants also agreed on the need to consider research agendas and activities in other areas to bridge knowledge gaps and increase added value in the field of health EDRM, while avoiding duplication with other areas.

In terms of the wide range of stakeholders involved in the WHO health EDRM research agenda development process, the participants classified stakeholders into four groups. The first group represented scientists and academics. They emphasize that the major role of scientists was to produce high-quality research and those of academics was to provide high-quality education and training to human resources (especially young researchers) in the field of health EDRM. It was also suggested that scientific expertise closely related to health EDRM includes not only health science (such as epidemiology, public health, medicine, nursing, etc.) but also social science (such as communication, economics, etc.). As a second group, the participants identified several users of the research outputs, including policymakers, government officials, front-line workers, hospital administrators and practitioners, as well as non-governmental organizations and civil society organizations. The third group is funding providers, including public and private research foundations, the media and publishers. The last group included research assistants, including database administrators, network service providers and language translators.

In addition, at the 2nd Core Group Meeting held during the COVID-19 pandemic, the participants highlighted the need for evidence and research focused on emergency preparedness, IHR (2005), health security and pandemic preparedness to be included as research themes and priorities. Research themes and questions will be further examined in the future and will be finalized on the basis of rigorous consultative and participatory processes.

Finally, the participants agreed on the following process to develop the WHO health EDRM research agenda [9], taking into account existing WHO and global research activities as well as priorities [10] and WHO guidance on research strategies and priority-setting [11]. They recognized that this process needs to be framed within a broader global health EDRM research strategy.

1. Gather information on existing health EDRM research programs and agendas.
2. Finalize criteria for prioritizing research themes and questions (preliminary criteria are displayed above).
3. Finalize a list of stakeholders to be consulted.
4. Conduct surveys and other consultative methods with key stakeholders to identify research needs.
5. Establish a process and working groups for reviewing results of surveys.
6. Draft a research agenda to address identified needs under each theme (preliminary research themes and questions are presented in Table 1).
7. Conduct a peer-review through global consultation.
8. Publish and disseminate finalized research agenda.
9. Monitoring progress and update research agenda every two years.

(Note that this list was adapted from a report of the 1st Core Group Meeting issued by the WHO Kobe Centre [9].)

### 3.2. WHO Health EDRM Knowledge Hub

The WHO health EDRM knowledge hub was the main subject of the 2nd Core Group Meeting. It should provide up-to-date knowledge and comprehensive information on health EDRM and is expected to serve as the main infrastructure for global hazard information. There are existing knowledge hubs that have already been operated in various research fields (Global Health Network, Cochrane, Campbell Collaboration, What Works Network, European Commission, NICE, Evidence Aid, etc.), and among them, the participants agreed that it was particularly important to collaborate with the climate change field, where there is mutual interaction with health EDRM and where synergy can be expected. An intergovernmental technical group established by the World Meteorological Organization (WMO), which focuses on identifying research priorities on climate change and related issues, launched the Health and Climate Science Portal in 2020, in a joint project with WHO. The participants agreed that the health EDRM RN would work with the WMO technical group.

### 3.3. Knowledge Hub for Information Sharing

The participants agreed on the following four steps for how this knowledge hub will expand the network. First, the WHO Kobe Centre will build an online web-based platform that serves as an information-sharing hub for the health EDRM RN. Second, this platform will then be used to exchange information on relevant health EDRM evidence and research results from the health EDRM RN and other sources. New research funding opportunities for health EDRM will also be identified on a regular basis and disseminated in a timely manner. Third, a listserve and other communication mechanisms will be established to expand the platform’s reach and form new networks. Fourth, events and spaces will be developed on the platform (e.g., webinars, moderated discussions, etc.) for collaboration, communication and engagement among health EDRM RN participants, policymakers, practitioners, other users, supporters and the broader community.

## 4. Follow-Up Actions

With regard to the health EDRM RN research agenda, after further review of criteria for developing a research agenda including the rationale of each criterion, in line with the agreed process described above, a working group will be formed to identify research priorities and to design a survey for stakeholder input. Discussions on the structure of the group will be held in due course. The research theme will be to capture lessons from COVID-19 with attention to IHR and emergency continuum including prevention, preparedness, response and recovery. The WHO Kobe Centre will build a website for the WHO health EDRM knowledge hub.

## 5. Conclusions

The objectives of the core group meetings were achieved through the participation of key stakeholders from the WHO headquarters and all WHO regional offices and external experts, the integration of their input and advice to the WHO on the health EDRM RN, agreement on the WHO health EDRM research agenda development process and the establishment of the WHO health EDRM knowledge hub. A consensus was also reached on additional activities that would benefit countries by increasing regional participation, including member countries and other key stakeholders, and promoting regional cooperation. The activities of the health EDRM RN are expected to be accelerated by advancing agreed processes and follow-up actions and paying attention to all-hazard emergency preparedness, to support the implementation of the IHR (2005) and health emergency and disaster risk management. This is pertinent in the context of COVID-19, concurrent events and risks to health such as climate change.

## Figures and Tables

**Table 1:** Preliminary research themes and questions (WHO Centre for Health Development, 2021).

	Theme	Additional Research Questions Proposed at the 1st Core Group Meeting [9]	Research Questions Proposed at the 2018 Kobe Expert Meeting [8]
Area 1 *	Health data management before, during and after emergencies and disasters	a. How can data on non-death health outcomes be captured? b. How can data be captured on non-communicable diseases (NCDs) in emergencies? c. How can data on long-term physical health and wellbeing and mortality risks be captured? d. How can data be more effectively used in health emergency risk profiling? e. How can data be used to conduct monitoring and evaluation more effectively? f. How can big data be used for improvements to the policy and practice of health emergency and disaster risk management (EDRM)?	a. What are the national and regional challenges inhibiting the implementation of WHO standardized medical data collection systems after emergencies and disasters? b. What broader health-related data are needed to inform effective health emergency and disaster risk management (EDRM), i.e., community vulnerabilities, hospital functional status, infrastructure, lifelines and health workforce?
Area 2 *	Psychosocial management before, during and after emergencies and disasters	a. How can common or standardized countermeasures apply psychosocial management across all settings (natural hazard-related events, human-induced events, disease outbreaks, etc.)? b. How can psychosocial impacts be monitored effectively (e.g., post-traumatic stress disorder [PTSD])? c. How should underlying risks and impacts be framed? d. What wellbeing indicators can be used to evaluate resilience for emergencies and disasters?	a. How can mental health and psychosocial risk be classified using longitudinal and multi-centric studies? b. How can methods for screening, diagnosis and treatment of affected people be standardized across different settings? c. How can assets associated with greater community resilience be identified before, during and after a disaster?
Area 3 *	Community disaster risk management including disaster risk literacy and addressing the needs of subpopulations	a. No additional questions were proposed. Follow-up is required.	a. What architecture is needed to support research into health EDRM, including consensus among disciplines and ethics? b. How can research be better translated to policy and practice across different backgrounds and contexts? c. What kind of technology for information and data management and communication is needed for risk communication, emergency response and research design?
Area 4 *	Health workforce development for health EDRM	a. What are the indicators that evaluate training effectiveness? b. How can “occupational health and safety” be more effectively addressed in health EDRM workforce development?	a. How can different countries strengthen health EDRM through disaster risk management training programs, and what strategies will support retention, motivation and deployment of trained people? b. What are the best practices for sustaining the development of the local health workforce for health EDRM, fostering positive interactions between external support workers and the local workforce, and enabling the transition to recovery and post-event health EDRM? c. What is the common knowledge or what are the competencies required for health EDRM?
Area 5 *	Research methods and ethics	a. How can the ethical approval processes for health EDRM be accelerated? b. How can effective structures, mechanisms or frameworks for health EDRM research funding be established/strengthened?	a. What are the definitions of the research methods and technical terms for the health EDRM? b. How can impact evaluation methods for intervention and qualitative–quantitative mixed methods be standardized? c. How can the publication process for health EDRM research become more systematic and effective? d. What are the challenges and best practices in addressing national health system, cultural and religious issues before, during and after interventions?
Area 6	Translational research	How can successful mechanisms be promoted to facilitate the translation of research findings into policy and practice?	Not available.
Area 7	Complex health risk conceptualization	How can directions be communicated for conceptualizing the complex health risks associated with various emergencies and disasters?	Not available.
Area 8	Cross-disciplinary research agenda	a. How can overlapping research topics with other non-health sectors be identified? b. What are the possible lessons for effective multi-disciplinary collaboration?	Not available.

* Originally proposed at the 2018 Kobe Expert Meeting [8].
